# The present time of loneliness in alcohol addiction

**DOI:** 10.1192/j.eurpsy.2026.10884

**Published:** 2026-07-31

**Authors:** G. Dzhupanov

**Affiliations:** State Psychiatric Hospital for Treatment of Drug Addiction and Alcoholism, Sofia, Bulgaria

## Abstract

**Introduction:**

In an era of accelerated social time, we often hear the call to live now, fully immersed in the moment. Thus, a presentism seems to be pervasive, as part of the spirit of the times. Against this background, in terms of the experience of time in patients suffering from addictions, there is also a tendency towards presentism, which reaches an excess. This seems to disrupt experience of self in time and interpersonal relationships, leading to isolation and loneliness.

**Objectives:**

To outline some aspects of the experience of time in patients with alcohol dependence. How is the tendency towards hyperpresentification related to the experience of loneliness? Does the presentism of our epoque matter for this?

**Methods:**

Analysis of interviews with patients suffering from alcohol dependence based on descriptive phenomenology and a review of relevant literature.

**Results:**

Several mechanisms have been outlined by which presentist attitudes contribute to the generation of loneliness. This happens by erasing complexity and narrowing the horizon of the possible future. The desire for something here and now, in the absence of a clear life plan, difficulties in planning daily activities, complicates and interrupts many interpersonal relationships, people become increasingly alone. In periods of abstinence, the problem of what to do with time often arises, how to structure it becomes obstacle, given that connections from the past are often broken, and the horizon for new ones is narrowed. Mimetic desire in times of great connectivity (e.g. through social media) facilitates the spread of maladaptive models of experience, including those emphasizing the value of living here and now, models often devoid of existential authenticity, which as a consequence disrupt authentic communication, and thus pave the way for isolation and experiences of loneliness.

**Conclusions:**

Based on the lived experience of patients, a significant role of presentism and hyperpresentification on the experience of loneliness is highlighted through several mechanisms. It seems that the spirit of the times with its inherent presentism facilitates the emergence of this trend or is at least a supporting factor. Therapeutic methods aimed at restoring the inherent historicity of the person, the ability for realistic planning and alternative experience of the moment “here and now” in this light seem to be relevant.

**Disclosure of Interest:**

None Declared

